# Antennal Lobe Atlas of an Emerging Corn Pest, *Athetis dissimilis*

**DOI:** 10.3389/fnana.2020.00023

**Published:** 2020-05-28

**Authors:** Jun-Feng Dong, Nan-Ji Jiang, Xin-Cheng Zhao, Rui Tang

**Affiliations:** ^1^Forestry College, Henan University of Science and Technology, Luoyang, China; ^2^State Key Laboratory of Integrated Management of Pest Insects and Rodents, Institute of Zoology, Chinese Academy of Sciences, Beijing, China; ^3^Department of Entomology, College of Plant Protection, Henan Agricultural University, Zhengzhou, China; ^4^State Key Laboratory for Biology of Plant Diseases and Insect Pests, Institute of Plant Protection, Chinese Academy of Agricultural Sciences, Beijing, China

**Keywords:** antennal lobe, *Athetis dissimilis*, digital atlas, glomerulus, sexual dimorphism

## Abstract

Moths develop sophisticated olfactory systems to sense the airborne chemical cues from the environment. Understanding the structural basis in the neuronal center is a fundamental neuroethological step. Little is known about the emerging crop pest* Athetis dissimilis* with regard to its morphology or its neuronal organizations. Through antibody staining and digital 3D modeling, we re-constructed the primary olfactory center—the antennal lobe of *A. dissimilis*. In the antennal lobes 68.8 ± 3.1 male glomeruli and 70.8 ± 1.0 female glomeruli were identified with obvious sexual dimorphism. In particular, male adults of *A. dissimilis* contain a macroglomerular complex (MGC) that consists of three subunits, while the female lobe has four relatively enlarged glomeruli at the entrance of the antennal nerve. Glomeruli were later clustered with deviation and variance, and referring to reported olfactory related receptor family genes in seven different moth species, we found that glomerular counts of these insects are better related to the sum of odorant receptor and ionotropic receptor numbers, suggesting olfactory receptors and ionotropic receptors may both involved in olfaction of Noctuidae moths.

## Introduction

The olfactory system is one of the most important sensory features in insect species, which broadly involves behavioral decisions (Dweck et al., 2015; Ebrahim et al., 2015; Joseph and Carlson, 2015; Wan et al., 2019). Detection of the airborne cues in insects starts in olfactory receptor neurons (ORNs) housed in sensilla that are mostly located on the antennae (Keil and Steinbrecht, 1984). The axons of ORNs directly project to the antennal lobe (AL), where synaptic attachments are made with second-order neurons in sophisticated structures called the glomeruli (Stocker et al., 1990; Christensen and Hildebrand, 2002). Postsynaptic projections in glomeruli contain projection neurons and local neurons, where olfactory signals are concentrated or diluted into the protocerebrum (Homberg et al., 1989). Usually, innate behaviors are decided finally in the lateral horn, and learning and memory driving behaviors are processed in the mushroom body (Yang et al., 1995; Gupta and Stopfer, 2012). Thus, anatomic characterization of ALs is important for neuroethological studies to understand insect behavioral decision (Sato and Touhara, 2008; Bisch-Knaden et al., 2018).

*Athetis dissimilis* (Hampson; Lepidoptera: Noctuidae) is found in Asian countries including Japan, Korea, India, Philippines, and Indonesia (Dong et al., 2016). It has become an emerging corn pest since its discovery in 2012 in Shandong province, China (Li et al., 2014). Larvae of this species live under plant residues, making it difficult to control. Novel management strategies such as olfaction-based ecological trapping are in urgent demand. Recent works have reported fundamental information on sensillar morphology and chemosensory genes within this species (Dong et al., 2016; Song et al., 2018a,b; Liu et al., 2019). In addition, some other works also involved trials in terms of trapping method development (Guo et al., 2016; Kim et al., 2016). However, the initial information of the ALs in this species remains unknown.

In the current study, we first investigated the morphology of *A. dissimilis* in all life stages. Utilization of synaptic antibody staining provided the first digital atlas of ALs in this species. Gender-based special structures including the macroglomerular complex (MGC) in males and the large female glomeruli (LFGs) are identified and described. Later comparison and clustering analysis revealed differences between the genders in terms of variations of glomeruli. Finally, a correlation between glomeruli numbers and olfaction-related receptors was carried out to show involvement of receptor classes in olfactory reception of Noctuidae species.

## Materials and Methods

### Insects

This work used a laboratory colony of both genders of *A. dissimilis* adults that has been described in previous works (Dong et al., 2016). A wild-type strain was collected at the campus of Academy of Agriculture and Forestry (N34°38′5.35″, E112°27′58.15″) during June to July 2015. Rejuvenation was ensured once every 10 generations by backcrossing with freshly collected field strains. Insects were reared under conditions of 27 ± 1°C with 70 ± 5% relative humidity and maintaining a 16 h: 8 h light/dark cycle.

### Wholemount Labeling of Brain

Brains of *A. dissimilis* were prepared according to previous works (Zhao et al., 2016). Insects were decapitated and ALs were dissected in Ringer’s solution (Jiang et al., 2019) before transfer to 4% paraformaldehyde in 0.1 M phosphate-buffered saline (PBS, pH 7.4) to be fixed at 4°C overnight. Brains were then rinsed in PBS (4 × 15 min) and preincubated with 5% normal goat serum (NGS; Sigma, St. Louis, MO, USA) in 0.1 M PBS containing 0.5% Triton X-100 (PBST; 0.1 M, pH 7.4) at 4°C overnight. SYNORF1 (Developmental Studies Hybridoma Bank, University of Iowa; Klagges et al., 1996; Berg et al., 2002) primary antibody was used at a concentration of 1:100 (with 5% NGS in PBST) to stain the brains at 4°C for 5 days. The brains were later rinsed in PBS (6 × 20 min) and subsequently incubated with Cy2-conjugated anti-mouse (Invitrogen, Eugene, OR, USA; dilution 1:300 with 1% NGS in PBST) for 3 days at 4°C. Finally, the brains were washed in PBS (6 × 20 min) and then dehydrated 20 min for each concentration with ascending ethanol series (including 50%, 70%, 90%, 95%, and 100%) before being cleaned and mounted in methyl salicylate in a perforated aluminum slide with two glass coverslips.

### Confocal Image Acquisition, Glomeruli Identification, and Three-Dimensional Reconstructions

All image stacks were acquired with a confocal laser scanning microscope (LSM 780, META Zeiss, Jena, Germany) with a 10× objective (Plan-Neofluar 10×/0.3) on the anti-synapsin immunolabeled whole-mount preparations. An argon laser at 488 nm was used to excite the Cy2 dye. The resolution of the confocal images was set to 1,024 × 1,024 voxels and the section interval was set to 3 or 4 μm. Amira software (AMIRA 5.3, Visage Imaging, Fürth, Germany) was used as previously described to conduct segmentation and reconstruction of the digital atlas of the ALs. Parameters for later analysis were acquired with the TissueStatistics tool embedded in Amira.

### Statistical Analysis

A parametric test for comparing volumes and counts between genders was performed using two-way *t*-tests in SPSS (IBM SPSS Statistics 22.0.0, Chicago, IL, USA). Circos 0.69-9 plots were constructed using Circos (Krzywinski et al., 2009). A dendrogram was developed with the Median Method in Statgraphics Centurion XVII (Statpoint Technologies Inc., Warrenton, VA, USA).

## Results

### Morphology of *A. dissimilis* in Different Stages

**Figure 1:**
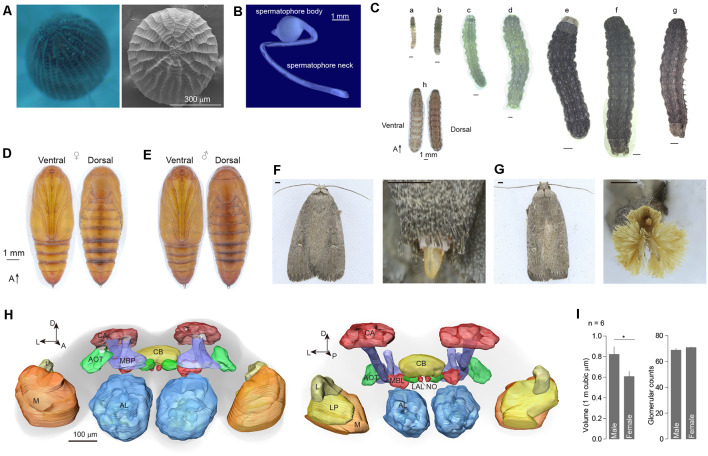
External morphology and brain structure of *Athetis dissimilis*. **(A)** Overview of anterior of eggs showing the micropylar area. **(B)** The spermatophore organ of males. **(C)** Morphology of different stages of larvae, which included 1st to 6th instars (a–f), matured larva (g), and overview (h). **(D)** Pupal morphology of females. **(E)** Pupal morphology of males. **(F)** Morphology of adults and copulation organs in female. Scale bar indicates 1 mm. **(G)** Morphology of adults and copulation organs in male. Scale bar indicates 1 mm. **(H)** Brain reconstruction of *A. dissimilis* from anterior (left) and posterior (right) views. Arrangement of neuropils: AL (antennal lobe), MBP (mushroom body peduncle), AOT (anterior optic tubercle), CA (calyx), CB (central complex), MBL (mushroom body lobe), LAL (lateral accessory lobe), NO (nodulus), L (lobula), LP (lobula plate), and M (medulla). **(I)** Comparison of single antennal lobe between male and female *A. dissimilis*. Asterisk indicates significant larger in volumes of male ALs than females (*t*-test, *t*_(10)_ = 2.39, *P* = 0.0381). No difference was observed in terms of glomerular counts between genders (*t*-test, *t*_(10)_ = 1.49, *P* = 0.166).

The egg of *A. dissimilis* is oval to nearly circular with a diameter of ~450 μm. Sides are truncated by a macropylar area at the anterior end and marked by a reticulate pattern of prominent longitudinal ridges joined by lesser cross ridges (Figure 1A). The male spermatophore body is ovoid and attached to a ~12 mm spermatophore neck (Figure 1B). Larva of *A. dissimilis* can develop to 6th instar before pupation (Figure 1C). A white dotted line exists along the longitude of the middle dorsal side of the larvae, paralleled with four symmetrical dotted stripes (Figure 1C). The pupa is light brown ventrally and dark reddish brown dorsally. Forewings extend to the fifth section of the abdomen (A5). An anterior row of short, stout, dorsal spines is present on segments A6–8. Sexual dimorphism presents ventrally on A9–10. The cremaster consists of a large pair of stout hooks arising dorsally from A10 (Figures 1D,E). Adults of both genders are moderately large, generally dark grayish moths with a small, white subapical spot on the forewing near the apex of the discal cell and a smaller black one at the anterior of the initial spot (Figures 1F,G). A female adult presents a spade-shaped ovipositor at the tip of the abdomen (Figure 1F), while the male has a phallic organ that is surrounded by a remarkable panicle of hairpencils (Figure 1G).

### Digital Atlas Showed Sexual Dimorphism Between Genders

A total of 12 brains of *A. dissimilis* were prepared (Supplementary Table S1). ALs within six brains from each gender were analyzed (Supplementary Tables S2, S3). The synaptic specific antibody staining resulted in an intense labeling of the ALs and the key neuropils including mushroom body peduncle, anterior optic tubercle, calyx, central complex, mushroom body lobe, lateral accessory lobe, nodulus, lobula, lobula plate, and medulla (Figure 1H). The total volumes of glomeruli were 822,516.77 ± 180,998.80 μm^3^ in males and 607,876.42 ± 125,467.75 μm^3^ in females, respectively (mean ± SD; Supplementary Table S1). Significantly larger ALs were observed in males than in females (Figure 1I). By manual segmentation and cross checking among specimens, we allocated a total of 68.8 ± 3.1 glomeruli in male AL and 70.8 ± 1.0 in female AL, respectively (mean ± SD; Supplementary Table S1). No difference in glomerular counts was observed between genders (Figure 1I).

**Figure 2:**
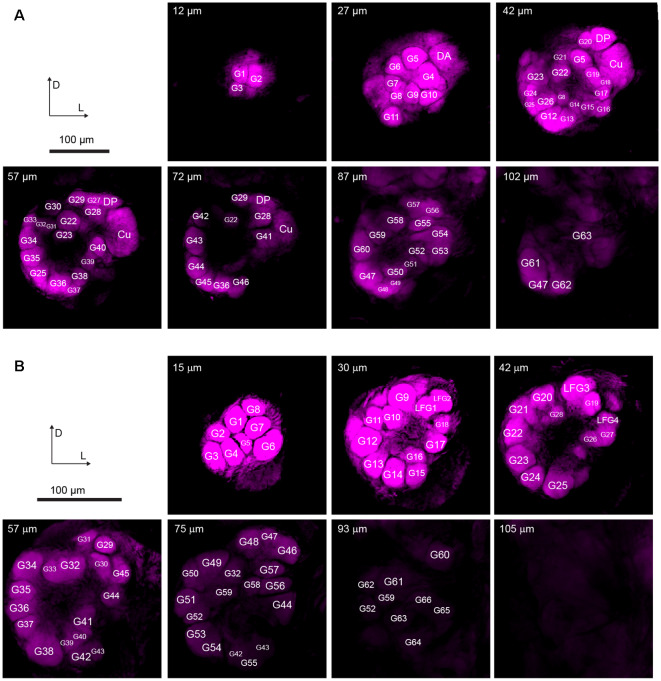
Confocal images showing arrangements of the antennal lobes in both sexes of *A. dissimilis*. **(A)** Organization of 66 glomeruli in male *A. dissimilis* (specimen 3) from the anterior view. Representative depths of 12 μm, 27 μm, 42 μm, 57 μm, 72 μm, 87 μm, and 102 μm are shown, respectively. Macroglomerular complex (MGC) area cluster is indicated by cumulus (CU), dorsal-anterior (DA), and dorsal-posterior (DP). Other ordinary glomeruli (G) are numbered and labeled among replicates. **(B)** Organization of 70 glomeruli in female *A. dissimilis* (specimen 1) from the anterior view. Representative depths of 15 μm, 30 μm, 42 μm, 57 μm, 75 μm, 93 μm, and 105 μm are shown, respectively. Large female glomerulus (LFG) area cluster is indicated by LFG 1–4. Other ordinary glomeruli (G) are numbered and labeled among replicates.

**Figure 3:**
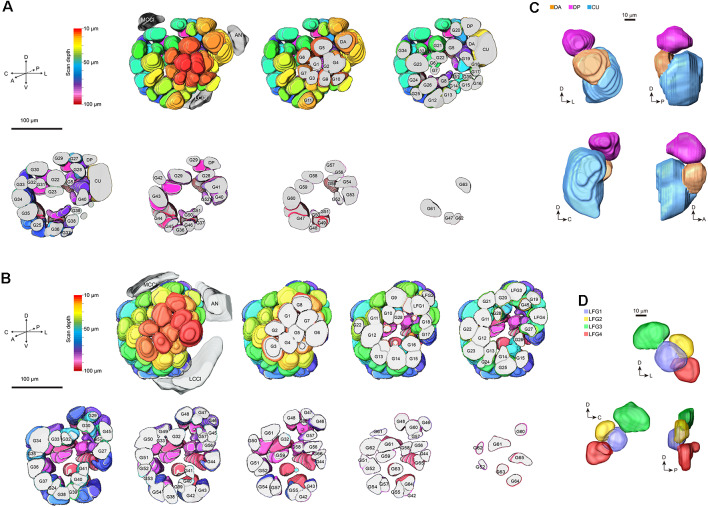
3-D modeling of antennal lobes in Figure 2. **(A)** Spatial organizations of glomeruli in male *A. dissimilis* by the anterior sectional view. Colors indicate scanning depth. Labeling of the glomeruli was the same as that in Figure 2. AN, antennal nerve; LCCl, the lateral cell cluster; MCCl, the medial cell cluster. **(B)** Spatial organizations of glomeruli in female *A. dissimilis* by anterior sectional view. Colors indicate scanning depth. Labeling of the glomeruli was the same as that in Figure 2. AN, antennal nerve; LCCl, the lateral cell cluster; MCCl, the medial cell cluster. **(C)** Anterior, posterior, and anteroposterior views of modeling of the MGC area in males, showing representative spatial organization of CU, DA, and DP, respectively. **(D)** Anterior, posterior, and anteroposterior views of modeling of the LFG area in females, showing representative spatial organization of LFG 1–4, respectively.

On the entry of male antenna, there are three enlarged glomeruli forming the MGC: the cumulus (CU), dorsal-anterior (DA), dorsal-posterior (DP; Figure 2A). The CU glomerulus in males has an outstanding volume that was significantly larger than that of any other glomeruli in the ALs. In females, four LFG subunits were located near the antennal nerve (Figure 2B) but did not exhibit remarkable enlargement in volumes. The border of each glomerulus was well captured and individual glomeruli could be identified with ease. Weak staining of the antennal and interglomerular nerve was observed, but no nerve tracts within the glomeruli were visible (Figure 2). All glomeruli were arrayed in a demarcated layer surrounding the hub (Figure 3). As the most anterior and prominent part of the deutocerebrum, ALs were surrounded by the medial cell cluster (MCCl) and the lateral cell cluster (LCCl), which were also strongly labeled yet with no glomerular organization (Figures 3A,B). Both MGCs and LFGs were identical in spatial allocations and relatively larger in size compared to other ordinary glomeruli (Figures 3C,D).

**Figure 4:**
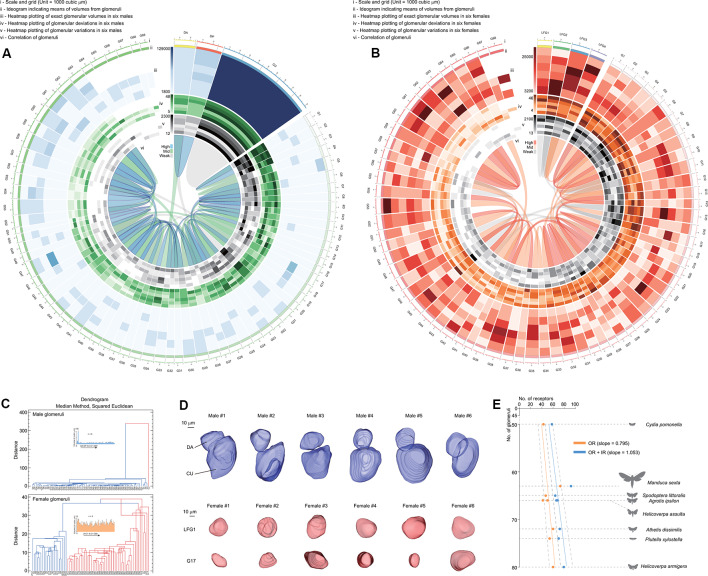
Statistics and analysis of the atlas results in *A. dissimilis*. **(A)** Overview of results in males including six replicates by Circos plot using data from Supplementary Table S2. Volume, deviation, and variation of glomeruli in all specimens are respectively shown with heat map circles. Width of the block indicates means of volumes in glomeruli. Blue ribbons indicate strong correlations of data pooled between glomeruli, and green and gray indicate medium and weak correlations. **(B)** Overview of results in females including six replicates by Circos plot using data from Supplementary Table S3. **(C)** Dendrograms showing clustering of glomeruli in both sexes pooled from all specimens. The median method using the squared Euclidean distance metric was utilized to analyze volumes, deviations, and variations among glomeruli. **(D)** Select glomeruli in both sexes and among specimens showing representative characters. DA and LFG1: glomeruli with constant volumes and shapes among replicates. CU and G17: glomeruli that formed distinct clusters with the previous ones, indicating that they were either larger in sizes or more distorted in shape/volume. **(E)** Exploration of relationships between olfaction-related receptors (odorant receptors, OR; ionotropic receptors, IR) and numbers of glomeruli in ALs in seven moth species. Orange indicates correlation of ORs against glomerular numbers, and blue indicates ORs + IRs against glomerular numbers in each species, respectively. A slope of 1.053 indicates that the sum of both ORs and IRs was better correlated with numbers of glomeruli in each species (linear regression, *t*_(7)_ = 18.5, *P* < 0.0001). Dotted lines indicate 95% CI.

The volume of a single glomerulus ranged from 5,139 ± 1,590 μm^3^ (G51) to 97,709 ± 19,605 μm^3^ (CU) in males and 5,156 ± 1,603 μm^3^ (G26) to 13,338 ± 2,995 μm^3^ (LFG3) in females, respectively (Supplementary Tables S2, S3). The volume, deviation (meaning the shape of glomeruli), and variation (meaning the consistency of glomeruli among individuals) of each glomerulus were determined using heatmaps of the Circos plot (Figures 4A,B; Krzywinski et al., 2009). Female glomeruli had higher variations in volume, while male glomeruli were relatively more consistent (Figures 4A,B). There were similar proportions of high-deviation glomeruli in ALs of both genders, indicating these glomeruli had various shapes (Figures 4A,B). When all parameters were assembled, several groups of glomeruli were highly correlated, lying in different clusters (Figure 4C). Cumulus formed a cluster distinguishable from other glomeruli, as it was higher in size (Figure 4D). Furthermore, glomerular clusters in *A. dissimilis* ALs showed different variations in terms of either size or shape; e.g., LFGs were relatively more identical in sizes and shapes while a separated cluster glomerulus G17 showed remarkable variations in sizes and shapes among individuals (Figures 4C,D).

## Discussion

### Antennal Lobe Morphological Conservation in Noctuidae

The moth antennal lobe is well known to be sexually dimorphic. We found that the arrangements of *A. dissimilis* antennal lobe is conserved within Noctuidae species. Male *A. dissimilis* moth shows three enlarged subunits in MGC of ALs and this three-part MGC arrangement is also similar to that of other reported species including *Helicoverpa armigera*, *H. assulta*, and *Mythimna separata* (Wu et al., 2015; Jiang et al., 2019). Specific glomeruli of MGC serving as projections are those of pheromone tuning ORNs and the major pheromone component generally projects to the largest subunit, CU (Hansson et al., 1992). To recognize pheromone more sensitively, male MGC show increasing size among olfactory glomeruli under high selection pressure (Hansson and Stensmyr, 2011). In contrast to the MGC, female LFGs were thought to be involved in encoding olfactory information of female-specific ovipositional behavior but more research is needed to confirm this. For *A. dissimilis*, our research offered a new perspective to understand how this pest senses olfactory cues. The three-part MGC arrangement of the male antennal lobe indicates that the sex pheromone of females may consist of two components. However, sex pheromones of female moths still need to be identified precisely. The ordinary glomeruli (OGs) have been reported to process plant odor information (Christensen and Hildebrand, 2002). In attempts to characterize the OGs in *A. dissimilis* functionally, further *in vivo* optical imaging or intracellular recording will be worthwhile to utilize in this pest.

### Olfactory Receptor Neurons and Ionotropic Receptor Neurons May Both Project to Antennal Lobes

In insects, ORNs expressing certain odorant receptor (ORs) that project from the antenna to the corresponding glomerulus (Vosshall and Stocker, 2007). We asked what kind of chemosensory receptors are involved by comparison between numbers of receptors and glomeruli as based on the one OR/one ORN rule (Vosshall et al., 2000). When comparing glomeruli with ORs in several reported moth species including *A. dissimilis* (Supplementary Table S4; Bengtsson et al., 2012; Jacquin-Joly et al., 2012; Liu et al., 2012, 2014; Poivet et al., 2013; Gu et al., 2014; Koenig et al., 2015; Xu et al., 2015; Zhang et al., 2015; Dong et al., 2016; Yang et al., 2017), the slope is 0.795, indicating that more glomeruli were not projected by ORs. The reason may be that other chemosensory receptor neurons may also be involved in projection to the ALs. Recent work has revealed that ionotropic receptors (IRs) are involved in olfaction in moths (Tang et al., 2020). When additional IRs were added to the correlation, we found a better slope of 1.053, meaning each receptor can project to its corresponding glomerulus (Figure 4E). This provided evidence that olfactory processes in moths involve both ORs and IRs at the periphery. The slope of 1.053 from ORs + IRs to glomeruli in *A. dissimilis* moths actually is not perfectly correlated, indicating that there are more receptor types than the number of glomeruli within one species. In *Drosophila*, it is reported that more than one IR may project to the same glomerulus in the ALs (Grabe et al., 2016). We thus speculated that a similar mechanism may also occur in moth species, indicating that several different IRs may project to the same glomerulus.

## Data Availability Statement

All datasets generated for this study are included in the article/Supplementary Material.

## Author Contributions

RT and X-CZ conceived the project. J-FD and X-CZ conducted the experiments. N-JJ and RT analyzed the data. RT drafted the manuscript with inputs from all.

## Conflict of Interest

The authors declare that the research was conducted in the absence of any commercial or financial relationships that could be construed as a potential conflict of interest.
