# Supplementary material for: Antennal Lobe Atlas of an Emerging Corn Pest, Athetis dissimilis
Source: Front Neuroanat. 2020 May 28;14:23. doi: 10.3389/fnana.2020.00023 (PMC7271962; doi:10.3389/fnana.2020.00023)
Supplement: Supplementary file 1 [file Data_Sheet_1.DOCX]

**Supplementary materials for:**

Antennal lobe atlas of an emerging corn pest *Athetis dissimilis*

Jun-Feng Dong^#^, Nan-Ji Jiang^#^, Xin-Cheng Zhao^*^, Rui Tang^*^

**List of contents:**

Table S1 – S4

**Table S1**. List of parameters of ALs in each gender of *A. dissimilis*. Highlighted specimens indicate the ones described in the text part.

| Specimen | Length with axis (μm) | | | volume (μm3) | Glomerular counts |
| --- | --- | --- | --- | --- | --- |
|  | x | y | z |  |  |
| Male#1 | 188.19 | 199.82 | 132 | 935757.0366 | 66 |
| Male#2 | 172.58 | 178.51 | 129 | 890007.7119 | 66 |
| Male#3 | 200.59 | 188.3 | 126 | 1093347.038 | 66 |
| Male#4 | 181.74 | 185.43 | 132 | 711644.4311 | 72 |
| Male#5 | 190.13 | 181.84 | 123 | 615103.3506 | 72 |
| Male#6 | 203.72 | 199.28 | 153 | 689241.0628 | 71 |
| Female#1 | 150.31 | 160.97 | 108 | 731819.6186 | 70 |
| Female#2 | 187.44 | 174.05 | 120 | 739149.6376 | 70 |
| Female#3 | 196.28 | 187.44 | 126 | 666362.1445 | 70 |
| Female#4 | 189.3 | 182.66 | 102 | 438445.7132 | 72 |
| Female#5 | 193.49 | 202.6 | 114 | 578059.4769 | 71 |
| Female#6 | 206.33 | 188.32 | 105 | 493421.977 | 72 |

**Table S2**. Glomeruli information of antennal lobes in male adults of *A. dissimilis*. Data was obtained by AMIRA 6.0. MGC (macro glomerular complex) is indicated by blue highlights. DA: dorsal anterior; DP: dorsal posterior; CU: cumulus.

| Glomeruli | Volume | | | | | | Deviation | | | | | | Variance | | | | | |
| --- | --- | --- | --- | --- | --- | --- | --- | --- | --- | --- | --- | --- | --- | --- | --- | --- | --- | --- |
|  | Male#1 | Male#2 | Male#3 | Male#4 | Male#5 | Male#6 | Male#1 | Male#2 | Male#3 | Male#4 | Male#5 | Male#6 | Male#1 | Male#2 | Male#3 | Male#4 | Male#5 | Male#6 |
| DA | 20576.63 | 18116 | 28329.64 | 23044.44 | 11366.69 | 23002.96 | 31.48443 | 16.95468 | 27.25896 | 9.352535 | 16.58357 | 26.3675 | 991.2694 | 287.4612 | 743.0507 | 87.46993 | 275.0146 | 695.2451 |
| DP | 20551.94 | 31579.97 | 32604.93 | 27469.77 | 15976.13 | 21407.67 | 20.98418 | 17.68856 | 30.28333 | 13.32605 | 14.82149 | 18.94015 | 440.3356 | 312.8852 | 917.0797 | 177.5837 | 219.6764 | 358.7292 |
| CU | 84803.16 | 104944.7 | 129374.8 | 97845.1 | 71249.63 | 98038.96 | 47.71014 | 23.20599 | 38.64061 | 12.22135 | 24.5061 | 36.52303 | 2276.258 | 538.518 | 1493.097 | 149.3615 | 600.5491 | 1333.932 |
| G1 | 11217.15 | 9020.063 | 12955.3 | 17051.59 | 8096.729 | 9225.577 | 41.99135 | 21.70667 | 45.31855 | 26.0033 | 33.79759 | 19.29024 | 1763.274 | 471.1794 | 2053.771 | 676.1717 | 1142.277 | 372.1134 |
| G2 | 14565.11 | 9951.117 | 13764.77 | 10060.7 | 5779.289 | 9178.367 | 44.67598 | 22.67795 | 47.55851 | 21.08018 | 28.26461 | 13.01879 | 1995.944 | 514.2894 | 2261.812 | 444.3739 | 798.8879 | 169.4889 |
| G3 | 6567.534 | 10035.66 | 8652.814 | 8476.873 | 6744.536 | 10734.32 | 35.81496 | 26.67911 | 47.7661 | 14.30653 | 33.49265 | 15.49246 | 1282.711 | 711.775 | 2281.6 | 204.6768 | 1121.757 | 240.0162 |
| G4 | 4707.885 | 6267.004 | 15427.8 | 19717.62 | 7715.613 | 8891.174 | 29.73844 | 16.44065 | 13.54052 | 19.60977 | 27.03654 | 7.670756 | 884.3748 | 270.295 | 183.3458 | 384.5431 | 730.9747 | 58.84051 |
| G5 | 6937.884 | 11388.34 | 35135.58 | 10009.17 | 6061.283 | 6951.639 | 34.64209 | 23.12663 | 30.6101 | 15.97063 | 29.50034 | 12.21608 | 1200.074 | 534.8411 | 936.9785 | 255.0609 | 870.27 | 149.2325 |
| G6 | 7786.231 | 11502.15 | 16980.15 | 9302.056 | 3668.044 | 16964.05 | 32.8478 | 26.14414 | 24.18907 | 17.90517 | 20.32002 | 18.09131 | 1078.978 | 683.5163 | 585.111 | 320.5951 | 412.9033 | 327.2956 |
| G7 | 11123.33 | 5529.964 | 11072.79 | 7844.777 | 3088.153 | 11725.73 | 30.05619 | 22.52593 | 25.52684 | 16.53363 | 25.98835 | 18.66916 | 903.3742 | 507.4177 | 651.6195 | 273.3608 | 675.3945 | 348.5375 |
| G8 | 4845.161 | 6430.67 | 8763.495 | 11641.27 | 6817.154 | 9068.211 | 30.97805 | 21.17002 | 26.16063 | 16.57533 | 19.84329 | 23.49046 | 959.6395 | 448.1698 | 684.3787 | 274.7416 | 393.7559 | 551.8016 |
| G9 | 8224.725 | 9772.277 | 13222.63 | 9434.476 | 7469.133 | 3430.577 | 27.19335 | 25.922 | 30.09658 | 18.79581 | 24.64157 | 18.61596 | 739.4785 | 671.9503 | 905.8038 | 353.2824 | 607.2071 | 346.5539 |
| G10 | 4087.673 | 6057.814 | 15051.68 | 5063.293 | 6714.852 | 10824.81 | 14.81816 | 16.36507 | 30.13148 | 14.13318 | 27.75046 | 10.27844 | 219.578 | 267.8156 | 907.9063 | 199.7468 | 770.0881 | 105.6464 |
| G11 | 8890.367 | 5872.471 | 14128.71 | 6729.313 | 7241.736 | 6640.842 | 24.28435 | 14.91053 | 26.29827 | 14.39072 | 27.41567 | 13.16735 | 589.7297 | 222.3239 | 691.5991 | 207.0929 | 751.6192 | 173.3791 |
| G12 | 5554.257 | 8753.428 | 15812.37 | 4589.71 | 12305.97 | 8468.253 | 26.5825 | 19.02386 | 22.71018 | 12.81641 | 25.40868 | 19.20082 | 706.6292 | 361.9073 | 515.7523 | 164.2603 | 645.6009 | 368.6715 |
| G13 | 3376.601 | 9733.257 | 13739.45 | 5598.194 | 7271.949 | 6906.397 | 11.61413 | 11.93901 | 27.27826 | 9.854448 | 31.89719 | 13.66219 | 134.888 | 142.5401 | 744.1034 | 97.11015 | 1017.431 | 186.6554 |
| G14 | 6190.271 | 3993.02 | 7282.433 | 8891.747 | 8054.854 | 8523.331 | 14.3969 | 9.45922 | 13.6982 | 14.35224 | 27.35834 | 24.7954 | 207.2707 | 89.47685 | 187.6407 | 205.9869 | 748.4788 | 614.8121 |
| G15 | 8026.218 | 7123.27 | 10603.8 | 4260.289 | 4833.125 | 8194.83 | 8.31487 | 20.32436 | 25.07781 | 10.51103 | 32.11356 | 21.27576 | 69.13706 | 413.0797 | 628.8964 | 110.4817 | 1031.281 | 452.6578 |
| G16 | 8548.658 | 8666.717 | 13403.65 | 3274.637 | 5545.001 | 4392.476 | 13.6684 | 24.71984 | 33.97434 | 19.55467 | 22.16635 | 16.28962 | 186.8252 | 611.0704 | 1154.256 | 382.385 | 491.3468 | 265.3516 |
| G17 | 34645.96 | 4174.028 | 5586.575 | 9401.86 | 4903.093 | 8889.207 | 18.16735 | 20.54439 | 30.14648 | 14.31105 | 20.17366 | 21.77795 | 330.0525 | 422.0718 | 908.8103 | 204.8061 | 406.9767 | 474.2793 |
| G18 | 24053.96 | 1786.237 | 7786.125 | 4021.541 | 6795.422 | 8108.279 | 15.56199 | 15.63616 | 18.62231 | 8.83198 | 15.14512 | 19.67068 | 242.1755 | 244.4895 | 346.7902 | 78.00387 | 229.3746 | 386.9354 |
| G19 | 13666.4 | 9697.489 | 10181.71 | 8527.754 | 6593.997 | 9713.411 | 25.06979 | 17.18633 | 20.31084 | 17.13911 | 21.14337 | 21.02047 | 628.4943 | 295.37 | 412.5301 | 293.7489 | 447.0421 | 441.8601 |
| G20 | 8729.388 | 7554.655 | 7144.551 | 11891.76 | 6730.754 | 4388.542 | 18.8829 | 16.32793 | 21.45083 | 19.68388 | 27.00399 | 17.50478 | 356.5639 | 266.6014 | 460.1383 | 387.4553 | 729.2155 | 306.4174 |
| G21 | 9128.378 | 5800.934 | 25209.94 | 15141.61 | 12439.01 | 10036.01 | 33.87461 | 20.45177 | 22.66096 | 19.66098 | 33.63903 | 33.75017 | 1147.489 | 418.275 | 513.519 | 386.5541 | 1131.584 | 1139.074 |
| G22 | 12061.55 | 5944.007 | 13678.48 | 11055.49 | 10530.78 | 7504.387 | 17.09463 | 20.16375 | 28.43303 | 13.28276 | 22.59551 | 18.24971 | 292.2265 | 406.5768 | 808.4373 | 176.4318 | 510.5571 | 333.0518 |
| G23 | 15810.47 | 8648.291 | 19332.59 | 11003.3 | 4847.436 | 5082.919 | 13.49455 | 20.11605 | 21.62557 | 19.71828 | 14.19039 | 19.50286 | 182.103 | 404.6553 | 467.6653 | 388.8105 | 201.3673 | 380.3617 |
| G24 | 9996.478 | 10997.06 | 11421.71 | 10866.97 | 8900.306 | 7227.03 | 39.78495 | 22.04117 | 24.19601 | 18.40218 | 26.06954 | 12.66636 | 1582.842 | 485.8133 | 585.4467 | 338.6404 | 679.621 | 160.4366 |
| G25 | 12402.27 | 10552.67 | 11292.27 | 13474.28 | 5296.401 | 8517.43 | 21.1708 | 18.05219 | 26.52818 | 16.75427 | 24.91902 | 18.78034 | 448.2026 | 325.8815 | 703.7441 | 280.7056 | 620.9577 | 352.7012 |
| G26 | 20638.85 | 15487.58 | 13838.87 | 11467.1 | 4384.16 | 3542.7 | 18.3953 | 18.70907 | 23.15246 | 16.88695 | 23.33793 | 17.46079 | 338.3871 | 350.0292 | 536.0366 | 285.1689 | 544.6591 | 304.8792 |
| G27 | 11939.09 | 14060.11 | 9286.884 | 2974.57 | 5008.576 | 4231.176 | 17.16295 | 11.22757 | 22.44777 | 9.13414 | 15.79866 | 23.2291 | 294.5667 | 126.0583 | 503.9023 | 83.43251 | 249.5977 | 539.591 |
| G28 | 21317.33 | 8155.125 | 14124.96 | 5151.356 | 5075.364 | 10028.14 | 9.264656 | 7.926556 | 17.62564 | 13.2588 | 21.84528 | 15.01267 | 85.83385 | 62.83028 | 310.663 | 175.7957 | 477.2164 | 225.3801 |
| G29 | 21318.31 | 13978.82 | 20979.67 | 3074.375 | 3148.581 | 5126.195 | 26.67357 | 8.815378 | 24.3154 | 9.697195 | 24.02428 | 9.475692 | 711.4791 | 77.71088 | 591.2388 | 94.03558 | 577.1661 | 89.78873 |
| G30 | 8645.442 | 18148.51 | 21121.31 | 10928.28 | 8211.753 | 2765.706 | 28.35655 | 15.23888 | 21.17103 | 14.70568 | 19.86143 | 22.87861 | 804.0941 | 232.2234 | 448.2126 | 216.2571 | 394.4765 | 523.4307 |
| G31 | 9401.943 | 6113.092 | 7856.474 | 7377.717 | 6188.498 | 6859.187 | 24.48893 | 10.53741 | 16.71967 | 11.78819 | 15.11767 | 24.83176 | 599.7078 | 111.037 | 279.5474 | 138.9615 | 228.544 | 616.6163 |
| G32 | 11443.31 | 16110.82 | 5057.558 | 2836.279 | 6998.966 | 3314.52 | 18.0489 | 11.33143 | 14.46747 | 14.5771 | 17.79545 | 27.95711 | 325.7628 | 128.4013 | 209.3078 | 212.4918 | 316.6779 | 781.5999 |
| G33 | 14721.15 | 12642.4 | 14345.38 | 4436.415 | 7719.324 | 5478.301 | 17.42161 | 12.8354 | 26.93228 | 7.192796 | 13.59895 | 25.88249 | 303.5125 | 164.7474 | 725.3479 | 51.73632 | 184.9315 | 669.9031 |
| G34 | 14687.57 | 23954.87 | 12180.54 | 3808.233 | 5101.867 | 9386.877 | 40.15549 | 21.34996 | 20.3309 | 10.19497 | 15.72654 | 27.35093 | 1612.464 | 455.8208 | 413.3455 | 103.9374 | 247.3241 | 748.0735 |
| G35 | 8525.943 | 11667.99 | 17849.65 | 4425.326 | 14725.71 | 3379.433 | 24.46435 | 17.1804 | 27.23384 | 9.508678 | 14.64223 | 28.63683 | 598.5045 | 295.1661 | 741.6819 | 90.41498 | 214.3948 | 820.0681 |
| G36 | 10224.61 | 6312.527 | 14896.91 | 7043.078 | 8438.621 | 4760.319 | 27.5411 | 17.45428 | 24.80484 | 10.42423 | 14.72268 | 19.54683 | 758.5124 | 304.6519 | 615.2799 | 108.6646 | 216.7574 | 382.0783 |
| G37 | 12530.66 | 6063.234 | 6781.555 | 8707.794 | 8679.8 | 10388.12 | 23.13136 | 14.22338 | 26.24294 | 16.54023 | 12.56999 | 19.84518 | 535.0599 | 202.3045 | 688.692 | 273.5792 | 158.0047 | 393.8311 |
| G38 | 11000.87 | 13158.32 | 9908.761 | 8084.83 | 7831.697 | 9976.999 | 14.29998 | 5.391003 | 21.10439 | 17.11627 | 15.03611 | 20.79873 | 204.4895 | 29.06291 | 445.3951 | 292.9668 | 226.0847 | 432.5873 |
| G39 | 11594.41 | 7550.319 | 4262.156 | 9234.214 | 10814.9 | 9085.914 | 11.0201 | 5.397582 | 18.09674 | 12.75478 | 15.54797 | 15.48123 | 121.4426 | 29.13389 | 327.4918 | 162.6843 | 241.7394 | 239.6685 |
| G40 | 10672 | 23766.27 | 11738.75 | 2341.822 | 12777.73 | 6526.751 | 8.693036 | 9.036894 | 21.51868 | 7.236963 | 22.25285 | 25.01997 | 75.56888 | 81.66545 | 463.0538 | 52.37363 | 495.1893 | 625.999 |
| G41 | 12900.02 | 21936.68 | 21321.1 | 3540.782 | 4957.69 | 8106.312 | 8.713004 | 8.555774 | 14.02132 | 9.899449 | 21.16387 | 15.80489 | 75.91644 | 73.20126 | 196.5975 | 97.9991 | 447.9096 | 249.7946 |
| G42 | 14740.9 | 8556.161 | 11788.46 | 5642.551 | 5394.462 | 11182.82 | 20.83078 | 9.58265 | 18.36639 | 9.052903 | 14.25508 | 22.20591 | 433.9215 | 91.82719 | 337.3241 | 81.95505 | 203.2074 | 493.1026 |
| G43 | 17269.16 | 12412.61 | 23281.46 | 8940.019 | 4828.884 | 5407.486 | 24.81559 | 15.88152 | 19.58472 | 12.42396 | 11.22518 | 25.11402 | 615.8135 | 252.2226 | 383.5611 | 154.3548 | 126.0047 | 630.7139 |
| G44 | 9512.554 | 11442.54 | 16882.6 | 3071.766 | 3768.226 | 10293.7 | 26.6427 | 12.2866 | 21.97229 | 14.6479 | 19.44715 | 21.73784 | 709.8336 | 150.9606 | 482.7816 | 214.5611 | 378.1915 | 472.5335 |
| G45 | 7412.919 | 10412.85 | 17950.02 | 3818.67 | 6683.048 | 15486.77 | 30.78373 | 11.04909 | 29.24539 | 16.53852 | 10.07365 | 21.4251 | 947.6382 | 122.0823 | 855.2925 | 273.5226 | 101.4784 | 459.035 |
| G46 | 14735.97 | 10506.06 | 10496.87 | 3609.928 | 7605.36 | 5954.333 | 22.38864 | 12.51652 | 24.70574 | 18.71713 | 10.07909 | 26.33436 | 501.251 | 156.6633 | 610.3737 | 350.3308 | 101.588 | 693.4983 |
| G47 | 42098.39 | 8194.145 | 19354.16 | 11995.48 | 3251.943 | 7445.375 | 16.74906 | 8.016548 | 19.04318 | 18.69003 | 5.667116 | 18.92196 | 280.5311 | 64.26505 | 362.6425 | 349.3173 | 32.11621 | 358.0405 |
| G48 | 10084.37 | 13139.9 | 8011.239 | 5538.18 | 4589.295 | 4833.101 | 8.800331 | 14.20513 | 24.50341 | 10.79066 | 8.00711 | 18.5763 | 77.44582 | 201.7858 | 600.4168 | 116.4384 | 64.1138 | 345.079 |
| G49 | 15437.16 | 11556.35 | 6609.906 | 7020.899 | 11726.61 | 5360.276 | 8.513688 | 12.04985 | 22.42263 | 13.15511 | 8.836642 | 19.32862 | 72.48288 | 145.199 | 502.7744 | 173.0568 | 78.08624 | 373.5956 |
| G50 | 5286.618 | 14810.16 | 9470.727 | 6772.366 | 4527.278 | 4659.998 | 5.559956 | 19.51674 | 12.98317 | 9.276989 | 10.14589 | 23.78024 | 30.91311 | 380.9031 | 168.5626 | 86.06253 | 102.9392 | 565.4999 |
| G51 | 6266.316 | 2924.313 | 6492.659 | 6125.267 | 5708.26 | 3314.52 | 4.935863 | 18.86028 | 12.28478 | 6.591769 | 4.584467 | 19.48937 | 24.36275 | 355.7101 | 150.9159 | 43.45142 | 21.01734 | 379.8355 |
| G52 | 16266.74 | 13629.81 | 9555.145 | 8063.304 | 8319.356 | 8373.834 | 9.033916 | 5.823057 | 13.61557 | 13.49749 | 7.565891 | 19.75634 | 81.61163 | 33.90799 | 185.3838 | 182.1821 | 57.24271 | 390.3131 |
| G53 | 18398.97 | 20867.97 | 28354.97 | 2313.12 | 10628.85 | 7683.391 | 8.618978 | 7.152124 | 13.81737 | 11.6362 | 7.931548 | 24.46284 | 74.28677 | 51.15287 | 190.9196 | 135.4011 | 62.90946 | 598.4305 |
| G54 | 14460.43 | 27588.04 | 10983.68 | 6750.187 | 6277.019 | 8423.011 | 9.43597 | 3.458869 | 12.06675 | 6.723851 | 5.238247 | 11.2062 | 89.03754 | 11.96378 | 145.6063 | 45.21018 | 27.43923 | 125.579 |
| G55 | 16408.96 | 11602.95 | 11361.68 | 6224.419 | 4688.947 | 12142.75 | 6.542675 | 7.481396 | 14.95483 | 5.496903 | 6.084037 | 20.83675 | 42.80659 | 55.97128 | 223.6469 | 30.21595 | 37.0155 | 434.17 |
| G56 | 9985.614 | 7923.175 | 11773.45 | 5947.184 | 9368.353 | 8541.035 | 8.615402 | 7.36985 | 20.38641 | 7.117795 | 6.987856 | 26.77734 | 74.22515 | 54.31469 | 415.6055 | 50.663 | 48.83014 | 717.0262 |
| G57 | 17559.51 | 24149.97 | 15322.75 | 12242.71 | 7150.565 | 8598.08 | 10.60174 | 11.3109 | 14.19805 | 13.96766 | 6.222773 | 20.84623 | 112.3968 | 127.9365 | 201.5847 | 195.0955 | 38.7229 | 434.5654 |
| G58 | 24473.69 | 17657.52 | 20887.75 | 8280.525 | 8405.227 | 8216.468 | 13.67412 | 10.08556 | 13.70841 | 10.01343 | 9.183997 | 15.72858 | 186.9817 | 101.7185 | 187.9206 | 100.2689 | 84.34581 | 247.3881 |
| G59 | 18028.62 | 14325.66 | 15591.01 | 8523.188 | 6130.191 | 7775.843 | 12.3471 | 11.87944 | 10.08044 | 15.2684 | 12.91408 | 22.04118 | 152.4508 | 141.1211 | 101.6153 | 233.124 | 166.7734 | 485.8136 |
| G60 | 21736.07 | 18833.53 | 22835.92 | 9043.085 | 11049.72 | 10401.89 | 14.14706 | 7.976519 | 16.70302 | 14.07543 | 7.612977 | 21.84087 | 200.1392 | 63.62486 | 278.991 | 198.1177 | 57.95741 | 477.0234 |
| G61 | 10531.76 | 10040 | 41653.57 | 8883.267 | 20181.13 | 12532.23 | 10.63042 | 9.836014 | 16.50695 | 18.30746 | 14.84978 | 16.29348 | 113.0058 | 96.74716 | 272.4794 | 335.1629 | 220.5161 | 265.4775 |
| G62 | 12690.65 | 23760.85 | 23467.18 | 7026.77 | 10267.34 | 9481.296 | 7.603944 | 12.16893 | 17.64406 | 15.68185 | 4.754961 | 24.61595 | 57.81997 | 148.0828 | 311.3128 | 245.9205 | 22.60965 | 605.9451 |
| G63 | 13734.54 | 26673.24 | 17730.53 | 5887.823 | 11740.39 | 6196.283 | 4.059089 | 7.082961 | 9.3072 | 15.05639 | 6.71931 | 15.18741 | 16.4762 | 50.16834 | 86.62398 | 226.695 | 45.14913 | 230.6574 |
| G64 |  |  |  | 7310.529 | 9158.448 | 11334.28 |  |  |  | 19.64654 | 5.723075 | 14.02714 |  |  |  | 385.9866 | 32.75359 | 196.7608 |
| G65 |  |  |  | 17221.2 | 12761.29 | 12026.69 |  |  |  | 10.13142 | 5.173286 | 11.66629 |  |  |  | 102.6457 | 26.76288 | 136.1022 |
| G66 |  |  |  | 16494.51 | 7981.175 | 7370.626 |  |  |  | 8.931447 | 5.784513 | 19.31147 |  |  |  | 79.77074 | 33.46059 | 372.9329 |
| G67 |  |  |  | 17178.14 | 9454.223 | 14078.35 |  |  |  | 6.925912 | 6.172221 | 18.05333 |  |  |  | 47.96826 | 38.09631 | 325.9227 |
| G68 |  |  |  | 11724.11 | 4323.733 | 7132.61 |  |  |  | 3.599998 | 3.581156 | 15.05362 |  |  |  | 12.95999 | 12.82468 | 226.6114 |
| G69 |  |  |  | 8873.483 | 6033.72 |  |  |  |  | 13.64516 | 3.530797 |  |  |  |  | 186.1904 | 12.46653 |  |

**Table S3**. Glomeruli information of antennal lobes in female adults of *A. dissimilis*. Data was obtained by AMIRA 6.0. LFG (large female glomerulus) area was highlighted in blue.

| Glomeruli | Volume | | | | | | Deviation | | | | | | Variance | | | | | |
| --- | --- | --- | --- | --- | --- | --- | --- | --- | --- | --- | --- | --- | --- | --- | --- | --- | --- | --- |
|  | Female#1 | Female#2 | Female#3 | Female#4 | Female#5 | Female#6 | Female#1 | Female#2 | Female#3 | Female#4 | Female#5 | Female#6 | Female#1 | Female#2 | Female#3 | Female#4 | Female#5 | Female#6 |
| LFG1 | 9326.827 | 11022.5 | 11598.78 | 6743.765 | 12047.41 | 12905.97 | 27.18786 | 42.0017 | 17.17439 | 11.81094 | 6.430002 | 21.60785 | 739.1796 | 1764.143 | 294.9597 | 139.4984 | 41.34492 | 466.8992 |
| LFG2 | 5549.774 | 14257.47 | 10133.95 | 8196.268 | 17233.33 | 17974.62 | 35.58607 | 35.14688 | 16.614 | 16.14027 | 9.052683 | 27.85729 | 1266.368 | 1235.303 | 276.025 | 260.5084 | 81.95107 | 776.0288 |
| LFG3 | 13386.71 | 13595.4 | 17475.06 | 11062.54 | 15428.87 | 9081.976 | 31.1279 | 30.2967 | 21.16856 | 12.8565 | 10.67303 | 26.11379 | 968.9462 | 917.8899 | 448.1078 | 165.2896 | 113.9135 | 681.9299 |
| LFG4 | 9498.309 | 21210.52 | 12988.83 | 5674.446 | 12453.03 | 8476.718 | 33.80241 | 24.37662 | 16.25584 | 15.68672 | 10.19813 | 17.07182 | 1142.603 | 594.2194 | 264.2525 | 246.0731 | 104.002 | 291.447 |
| G1 | 10449.25 | 7191.662 | 10376.32 | 6104.664 | 11315.77 | 11083.98 | 25.40823 | 45.65215 | 24.27269 | 14.64084 | 18.05353 | 26.95331 | 645.578 | 2084.119 | 589.1636 | 214.3543 | 325.93 | 726.4809 |
| G2 | 7024.072 | 12844.17 | 4998.558 | 5930.363 | 11592.5 | 8473.614 | 22.79724 | 41.8226 | 23.59964 | 12.29248 | 12.45542 | 21.00978 | 519.714 | 1749.13 | 556.943 | 151.1051 | 155.1375 | 441.4107 |
| G3 | 9901.402 | 8916.324 | 6049.714 | 6176.597 | 13434.87 | 9600.326 | 15.16411 | 35.02123 | 14.81994 | 12.54446 | 10.82418 | 30.46123 | 229.9502 | 1226.486 | 219.6305 | 157.3635 | 117.1628 | 927.8863 |
| G4 | 7981.697 | 7149.709 | 7331.042 | 6545.948 | 9287.649 | 7750.408 | 17.80713 | 32.21385 | 22.49488 | 14.30488 | 10.15194 | 30.72087 | 317.094 | 1037.732 | 506.0194 | 204.6296 | 103.0619 | 943.7716 |
| G5 | 5790.294 | 7263.769 | 7369.227 | 6166.914 | 6126.057 | 8898.847 | 37.7157 | 39.95808 | 23.85883 | 14.67383 | 12.15439 | 31.51148 | 1422.474 | 1596.648 | 569.2439 | 215.3214 | 147.7293 | 992.9735 |
| G6 | 17096.96 | 10505.29 | 10226.23 | 5946.963 | 8116.268 | 9072.665 | 30.62742 | 43.23203 | 27.16326 | 13.36948 | 12.06018 | 28.32291 | 938.039 | 1869.009 | 737.8425 | 178.743 | 145.448 | 802.1873 |
| G7 | 9055.128 | 8646.907 | 5133.798 | 7673.367 | 7013.123 | 4870 | 29.2623 | 45.47777 | 20.15903 | 14.89602 | 11.80855 | 25.96284 | 856.2822 | 2068.227 | 406.3866 | 221.8915 | 139.4419 | 674.0692 |
| G8 | 8578.543 | 9427.626 | 6578.474 | 4271.743 | 7706.853 | 3414.277 | 25.75094 | 36.55745 | 22.76042 | 8.59303 | 13.16182 | 13.79385 | 663.1108 | 1336.448 | 518.0369 | 73.84016 | 173.2334 | 190.2702 |
| G9 | 9745.51 | 9118.223 | 15558.38 | 5935.897 | 7346.72 | 7908.707 | 16.02782 | 35.12058 | 17.47526 | 11.56354 | 16.08044 | 32.85529 | 256.8912 | 1233.455 | 305.3846 | 133.7154 | 258.5807 | 1079.47 |
| G10 | 5549.774 | 9082.825 | 14106.28 | 9178.437 | 5409.582 | 4565.819 | 20.78082 | 39.83005 | 19.54212 | 18.63913 | 9.469453 | 30.22586 | 431.8425 | 1586.433 | 381.8943 | 347.4172 | 89.67054 | 913.6029 |
| G11 | 8304.617 | 10800.93 | 15261.38 | 9207.487 | 5849.323 | 7384.15 | 23.11779 | 23.52236 | 19.24153 | 11.15288 | 12.24164 | 24.75137 | 534.4324 | 553.3013 | 370.2366 | 124.3868 | 149.8577 | 612.6304 |
| G12 | 14092.68 | 11311.58 | 8607.066 | 7025.966 | 4810.623 | 7508.305 | 28.34815 | 36.18152 | 16.71893 | 8.572741 | 14.01135 | 28.23587 | 803.6174 | 1309.103 | 279.5228 | 73.49188 | 196.3178 | 797.2644 |
| G13 | 8048.508 | 6973.376 | 12355.06 | 5082.378 | 7566.591 | 7654.188 | 31.31594 | 35.90744 | 8.369426 | 11.15154 | 10.10569 | 26.06529 | 980.6883 | 1289.344 | 70.04729 | 124.3568 | 102.125 | 679.3993 |
| G14 | 12224.2 | 6323.104 | 4653.83 | 5855.663 | 4969.84 | 5009.675 | 30.28872 | 38.19098 | 16.17936 | 17.29814 | 11.84823 | 20.35458 | 917.4067 | 1458.551 | 261.7717 | 299.2257 | 140.3804 | 414.3088 |
| G15 | 10776.63 | 8370.28 | 13371.74 | 12773.73 | 10114.06 | 4413.729 | 33.55159 | 29.16092 | 27.39475 | 16.71148 | 15.71798 | 18.00867 | 1125.709 | 850.3591 | 750.4722 | 279.2734 | 247.055 | 324.3121 |
| G16 | 10565.06 | 5746.25 | 6136.161 | 3672.758 | 9177.713 | 10162.13 | 31.44831 | 34.40009 | 24.3215 | 15.28898 | 9.764688 | 24.75119 | 988.9959 | 1183.366 | 591.5355 | 233.7528 | 95.34911 | 612.6216 |
| G17 | 3583.301 | 8213.611 | 16266.4 | 4559.477 | 3328.39 | 9280.625 | 33.83153 | 42.48757 | 36.61996 | 14.27764 | 11.91931 | 23.26284 | 1144.573 | 1805.193 | 1341.021 | 203.8511 | 142.07 | 541.1599 |
| G18 | 4540.926 | 8382.079 | 6566.276 | 5613.579 | 6179.13 | 7182.397 | 24.08932 | 34.33959 | 22.83485 | 12.19553 | 9.439408 | 14.29877 | 580.2954 | 1179.207 | 521.4304 | 148.7311 | 89.10243 | 204.4549 |
| G19 | 8039.6 | 5251.336 | 4488.891 | 10055.47 | 4787.878 | 4274.054 | 25.34297 | 27.10522 | 21.1719 | 12.32632 | 8.959086 | 25.41185 | 642.2659 | 734.6931 | 448.2492 | 151.9383 | 80.26522 | 645.762 |
| G20 | 8228.898 | 5203.483 | 9029.756 | 7969.401 | 3643.033 | 5422.492 | 29.34903 | 22.04613 | 17.9468 | 14.32729 | 11.68881 | 27.31063 | 861.3657 | 486.032 | 322.0877 | 205.2711 | 136.6283 | 745.8706 |
| G21 | 12972.48 | 8588.566 | 12331.72 | 4450.193 | 2092.564 | 8889.535 | 24.49591 | 29.12096 | 18.23513 | 9.105983 | 10.44314 | 29.20532 | 600.0495 | 848.0303 | 332.5199 | 82.91892 | 109.0592 | 852.9508 |
| G22 | 12368.96 | 10769.47 | 13242.33 | 4968.944 | 5394.418 | 4870 | 26.26661 | 33.34387 | 16.30485 | 11.49517 | 10.27769 | 22.18013 | 689.9346 | 1111.814 | 265.8481 | 132.1388 | 105.6308 | 491.9581 |
| G23 | 9280.059 | 13531.81 | 19094.76 | 4096.059 | 7820.579 | 2594.85 | 20.16274 | 26.56892 | 16.4638 | 11.84155 | 12.67283 | 19.43292 | 406.5362 | 705.9075 | 271.0568 | 140.2222 | 160.6005 | 377.6385 |
| G24 | 8687.667 | 11447.93 | 10152.51 | 6529.348 | 7073.777 | 3914.003 | 24.79411 | 25.18483 | 19.6366 | 11.24716 | 10.80263 | 26.23812 | 614.7479 | 634.2755 | 385.5959 | 126.4985 | 116.6968 | 688.439 |
| G25 | 14502.46 | 13364.65 | 9711.257 | 4047.642 | 11782.05 | 5233.155 | 30.64526 | 34.57178 | 16.17851 | 8.729189 | 9.687923 | 19.55855 | 939.1321 | 1195.208 | 261.7443 | 76.19873 | 93.85587 | 382.537 |
| G26 | 4342.72 | 3803.302 | 7024.499 | 7243.15 | 4935.722 | 3588.095 | 24.69297 | 16.3159 | 19.46065 | 11.744 | 9.856498 | 17.2005 | 609.7426 | 266.2084 | 378.7167 | 137.9215 | 97.15054 | 295.857 |
| G27 | 13115.02 | 4414.898 | 7710.773 | 7001.066 | 5303.437 | 6161.218 | 37.32262 | 33.0434 | 26.49513 | 12.12162 | 11.18801 | 16.0195 | 1392.978 | 1091.867 | 701.9921 | 146.9338 | 125.1717 | 256.6245 |
| G28 | 4679.003 | 6450.274 | 7527.802 | 2802.64 | 7104.104 | 6080.517 | 23.11186 | 16.90932 | 23.26445 | 8.942119 | 13.14022 | 19.91894 | 534.1581 | 285.9252 | 541.2345 | 79.96149 | 172.6654 | 396.7642 |
| G29 | 6175.571 | 6041.888 | 5154.481 | 6637.248 | 8988.17 | 5214.532 | 32.13974 | 15.26471 | 28.16544 | 12.77594 | 13.16546 | 17.18623 | 1032.963 | 233.0114 | 793.2918 | 163.2245 | 173.3292 | 295.3665 |
| G30 | 4213.552 | 13444.63 | 6423.081 | 2878.723 | 8828.953 | 4069.197 | 23.50012 | 22.65878 | 26.98808 | 11.63064 | 12.26369 | 22.83427 | 552.2557 | 513.4205 | 728.3565 | 135.2717 | 150.3981 | 521.404 |
| G31 | 12827.73 | 11759.3 | 4575.868 | 9282.187 | 5614.289 | 3212.524 | 25.97449 | 16.17263 | 20.08607 | 12.88855 | 12.58859 | 18.8957 | 674.6743 | 261.554 | 403.4502 | 166.1146 | 158.4727 | 357.0476 |
| G32 | 14061.51 | 16018.18 | 10008.25 | 8871.336 | 5682.525 | 5469.051 | 25.30152 | 19.63334 | 14.3959 | 13.73541 | 13.61519 | 22.45202 | 640.1668 | 385.4681 | 207.242 | 188.6615 | 185.3735 | 504.0933 |
| G33 | 6961.715 | 8707.87 | 14936.81 | 6342.598 | 5674.943 | 5642.868 | 29.29433 | 24.50678 | 15.01249 | 8.924188 | 9.935288 | 19.73172 | 858.1578 | 600.5824 | 225.3749 | 79.64113 | 98.70995 | 389.3409 |
| G34 | 11589.5 | 6224.121 | 7622.204 | 4238.543 | 10413.54 | 4674.455 | 22.40951 | 18.40468 | 11.52232 | 8.81296 | 10.96501 | 15.5019 | 502.1861 | 338.7322 | 132.7638 | 77.66827 | 120.2314 | 240.3089 |
| G35 | 9709.877 | 10182.12 | 14257.43 | 10193.81 | 7418.746 | 6822.346 | 25.2115 | 13.56189 | 11.28504 | 13.18441 | 12.44983 | 18.43562 | 635.6199 | 183.925 | 127.3521 | 173.8287 | 154.9984 | 339.8721 |
| G36 | 10462.62 | 6531.558 | 7874.121 | 3735.008 | 4985.003 | 5661.492 | 27.30618 | 12.31193 | 9.167212 | 10.82592 | 9.315899 | 27.43635 | 745.6274 | 151.5835 | 84.03777 | 117.2006 | 86.78597 | 752.7535 |
| G37 | 11222.03 | 13019.85 | 14065.44 | 3087.607 | 3286.69 | 3886.068 | 26.85428 | 18.30347 | 15.78853 | 7.318473 | 10.598 | 13.65784 | 721.1522 | 335.0169 | 249.2776 | 53.56005 | 112.3177 | 186.5365 |
| G38 | 12705.24 | 17002.11 | 16290.26 | 9034.57 | 5489.19 | 7585.902 | 18.03116 | 29.37098 | 10.30413 | 10.08942 | 13.22769 | 13.45607 | 325.1226 | 862.6547 | 106.175 | 101.7965 | 174.9718 | 181.0658 |
| G39 | 3169.072 | 9130.022 | 14176.81 | 6761.748 | 8070.777 | 5869.452 | 14.15444 | 22.10902 | 20.9532 | 11.63471 | 11.74417 | 16.69932 | 200.3483 | 488.8087 | 439.0367 | 135.3664 | 137.9255 | 278.8672 |
| G40 | 7652.096 | 7066.459 | 4012.105 | 5901.313 | 5265.528 | 5329.376 | 22.68914 | 20.08379 | 3.852861 | 9.57117 | 11.48959 | 19.16374 | 514.7971 | 403.3587 | 14.84454 | 91.6073 | 132.0107 | 367.249 |
| G41 | 9342.416 | 15622.9 | 7361.272 | 3069.624 | 4871.277 | 3330.472 | 20.18773 | 26.10696 | 6.211879 | 8.369021 | 13.35962 | 16.18085 | 407.5446 | 681.5732 | 38.58744 | 70.04053 | 178.4794 | 261.82 |
| G42 | 19499.93 | 7031.717 | 7726.683 | 3313.091 | 8882.025 | 9749.312 | 14.70069 | 15.37061 | 19.45751 | 6.089372 | 10.05926 | 15.36293 | 216.1103 | 236.2555 | 378.5948 | 37.08045 | 101.1886 | 236.0197 |
| G43 | 10418.07 | 3700.386 | 10860.53 | 4992.461 | 5784.878 | 11568.19 | 14.83682 | 14.26154 | 21.48701 | 10.01012 | 7.774142 | 23.01903 | 220.1312 | 203.3915 | 461.6918 | 100.2024 | 60.43728 | 529.8758 |
| G44 | 13208.55 | 8446.975 | 11399.89 | 5359.045 | 7092.731 | 8678.471 | 15.06846 | 13.29385 | 13.14664 | 11.71796 | 10.15245 | 17.02833 | 227.0584 | 176.7264 | 172.8341 | 137.3106 | 103.0721 | 289.9639 |
| G45 | 14616.04 | 19683.82 | 7324.677 | 6460.181 | 11289.23 | 3799.159 | 36.22709 | 12.90461 | 25.24545 | 8.837593 | 12.11102 | 22.5378 | 1312.402 | 166.529 | 637.3326 | 78.10306 | 146.6767 | 507.9523 |
| G46 | 14426.74 | 10993 | 9220.152 | 3409.924 | 4674.151 | 9078.873 | 24.61418 | 7.342666 | 19.32668 | 9.778993 | 9.729248 | 25.78923 | 605.8577 | 53.91475 | 373.5207 | 95.62871 | 94.65827 | 665.0844 |
| G47 | 6605.389 | 6006.49 | 13410.99 | 6568.081 | 8836.535 | 9532.04 | 22.3437 | 12.37311 | 11.44501 | 9.082718 | 9.027865 | 21.18985 | 499.241 | 153.0939 | 130.9883 | 82.49577 | 81.50237 | 449.0096 |
| G48 | 10110.74 | 15115.54 | 12585.23 | 4895.628 | 10284.65 | 5754.608 | 17.0978 | 10.10375 | 21.22581 | 8.023666 | 9.352552 | 23.54172 | 292.3349 | 102.0857 | 450.5349 | 64.37921 | 87.47024 | 554.2127 |
| G49 | 12760.92 | 12648.83 | 3978.163 | 3484.624 | 10989.75 | 8209.784 | 21.775 | 9.373673 | 5.321487 | 10.41472 | 12.01827 | 18.7276 | 474.1504 | 87.86574 | 28.31823 | 108.4665 | 144.4387 | 350.7232 |
| G50 | 7253.456 | 8553.168 | 6637.873 | 3795.875 | 8173.131 | 8690.886 | 21.13862 | 9.943085 | 5.484767 | 6.367426 | 11.67573 | 22.36731 | 446.8412 | 98.86493 | 30.08267 | 40.54412 | 136.3226 | 500.2967 |
| G51 | 12647.34 | 9850.434 | 3764.431 | 5758.83 | 5932.723 | 3165.966 | 19.89064 | 11.05271 | 5.586016 | 8.576631 | 11.38891 | 15.30462 | 395.6374 | 122.1624 | 31.20357 | 73.55859 | 129.7073 | 234.2314 |
| G52 | 14493.55 | 11676.7 | 9656.631 | 8688.736 | 8878.234 | 9187.509 | 18.25098 | 14.91409 | 4.495186 | 11.45056 | 11.19253 | 16.66405 | 333.0983 | 222.43 | 20.2067 | 131.1153 | 125.2728 | 277.6905 |
| G53 | 25096.47 | 12404.32 | 8227.335 | 8423.135 | 8025.287 | 5236.259 | 25.90889 | 14.1649 | 5.536333 | 10.49747 | 7.182931 | 11.57474 | 671.2705 | 200.6443 | 30.65098 | 110.1968 | 51.59449 | 133.9746 |
| G54 | 11634.04 | 9521.365 | 7764.869 | 5193.045 | 6660.571 | 9839.325 | 17.80159 | 17.41174 | 7.642916 | 10.16707 | 11.04253 | 14.48343 | 316.8966 | 303.1686 | 58.41417 | 103.3694 | 121.9375 | 209.7696 |
| G55 | 7725.588 | 15700.26 | 8840.421 | 7367.65 | 8070.777 | 6642.32 | 7.258121 | 13.78992 | 12.74486 | 12.56398 | 6.352734 | 10.63749 | 52.68031 | 190.1619 | 162.4314 | 157.8536 | 40.35723 | 113.1563 |
| G56 | 14823.15 | 6027.467 | 6232.685 | 6936.049 | 6963.841 | 3588.095 | 23.35314 | 8.741114 | 10.84226 | 12.00707 | 10.26416 | 14.7697 | 545.3692 | 76.40706 | 117.5546 | 144.1696 | 105.353 | 218.144 |
| G57 | 9355.778 | 7596.771 | 9054.682 | 2477.556 | 7953.26 | 5649.076 | 24.10424 | 8.911947 | 13.77794 | 11.00563 | 7.756244 | 10.28396 | 581.0145 | 79.42281 | 189.8316 | 121.1239 | 60.15932 | 105.7599 |
| G58 | 7108.699 | 12100.82 | 10179.56 | 2756.989 | 14356.05 | 4336.132 | 11.8257 | 14.26687 | 6.845804 | 9.502208 | 5.41033 | 14.71996 | 139.8472 | 203.5437 | 46.86503 | 90.29195 | 29.27168 | 216.6773 |
| G59 | 11371.25 | 10605.59 | 9480.554 | 5819.696 | 10102.69 | 6633.009 | 9.41414 | 9.829495 | 7.631776 | 7.192774 | 8.411927 | 15.06628 | 88.62604 | 96.61899 | 58.244 | 51.736 | 70.76051 | 226.9928 |
| G60 | 14529.18 | 16100.78 | 11792.88 | 5713.179 | 10121.64 | 9575.495 | 15.68353 | 8.093149 | 8.442175 | 7.03018 | 4.998716 | 8.125588 | 245.9732 | 65.49906 | 71.27033 | 49.42343 | 24.98716 | 66.02516 |
| G61 | 18909.76 | 20494.04 | 5675.816 | 4321.543 | 9215.622 | 6772.684 | 14.78373 | 8.096245 | 7.324079 | 11.77243 | 4.072938 | 9.995431 | 218.5587 | 65.54918 | 53.64212 | 138.5901 | 16.58883 | 99.90865 |
| G62 | 16306.36 | 14527.54 | 9502.829 | 5068.545 | 8089.732 | 5875.66 | 17.57768 | 11.37103 | 6.908008 | 6.079221 | 6.982376 | 10.5908 | 308.9747 | 129.3003 | 47.72057 | 36.95693 | 48.75357 | 112.165 |
| G63 | 9262.243 | 21862.76 | 9449.263 | 4376.876 | 17358.43 | 4922.766 | 7.628991 | 9.913973 | 8.209711 | 9.611212 | 7.866688 | 5.927033 | 58.20151 | 98.28686 | 67.39936 | 92.3754 | 61.88478 | 35.12973 |
| G64 | 12689.65 | 9245.393 | 7791.917 | 6158.614 | 9204.25 | 4004.016 | 7.467291 | 7.651746 | 6.210973 | 7.661499 | 10.73313 | 12.49871 | 55.76044 | 58.54922 | 38.57619 | 58.69856 | 115.2 | 156.2177 |
| G65 | 6202.295 | 11567.89 | 8826.631 | 7128.333 | 11137.6 | 7759.72 | 15.75911 | 10.46974 | 4.381223 | 6.517052 | 9.295509 | 10.92631 | 248.3495 | 109.6155 | 19.19512 | 42.47197 | 86.4065 | 119.3843 |
| G66 | 13384.49 | 17757.26 | 6762.505 | 6837.832 | 10963.22 | 5447.324 | 7.635189 | 7.565167 | 3.650698 | 5.692651 | 12.40713 | 14.04453 | 58.2961 | 57.23175 | 13.3276 | 32.40627 | 153.9368 | 197.2489 |
| G67 |  |  |  | 3945.275 | 7983.587 | 9299.249 |  |  |  | 6.70379 | 5.903422 | 14.82814 |  |  |  | 44.9408 | 34.8504 | 219.8736 |
| G68 |  |  |  | 10597.74 |  | 9957.273 |  |  |  | 9.53507 |  | 10.67061 |  |  |  | 90.91754 |  | 113.8618 |

**Table S4**. List of information on glomeruli and olfaction related receptors by referring to reported works. Male glomeruli included the MGC complex.

| Family | Species | Male | Female | Reference | OR | IR | GR | Reference |
| --- | --- | --- | --- | --- | --- | --- | --- | --- |
| Noctuidae | *Athetis dissimilis* | 66-72 | 70-72 | current research | 60 | 12 | 18 | Dong et al., 2016 |
|  | *Spodoptera littoralis* | 67 | 29 or 55-63 | Sadek et al., 2002; Couton et al., 2009; Wan et al., 2015 | 47 | 17 | 6 | Jacquin-Joly et al., 2012; Poivet et al., 2013 |
|  | *Helicoverpa assulta* | 65 | 66 | Berge et al., 2002; Skiri et al., 2005 | 64 or 50 | 19 or 18 | 24 | Xu et al., 2015; Zhang et al., 2015 |
|  | *Helicoverpa armigera* | 65 or 70-80 | 65 or 81 | Skiri et al., 2005; Zhao et al., 2016a; Zhao et al., 2016b | 60 or 47 | 19 or 12 or 10 | 21 | Liu et al., 2012; Liu et al., 2014; Zhang et al., 2015 |
|  | *Agrotis ipsilon* | 66 | -* | Greiner et al., 2004 | 42 | 24 | 1 | Gu et al., 2014 |
| Plutellidae | *Plutella xylostella* | 74-76 | 74-77 | Yan et al., 2019 | 54 | 16 | 7 | Yang et al., 2017 |
| Tortricidae | *Cydia pomonella* | 50 | 49 | Trona et al., 2010 | 43 | 15 | 1 | Bengtsson et al., 2012 |
| Sphingidae | *Manduca sexta* | 63 | 63 | Rospars and Hildebrand, 2000 | 73 | 19 | 45 | Koenig et al., 2015; Grosse-Wilde et al., 2011 |

* Not reported.

**References:**

Bengtsson, J. M., Trona, F., Montagné, N., Anfora, G., Ignell, R., Witzgall, P., Jacquin-Joly, E. 2012. Putative chemosensory receptors of the codling moth, *Cydia pomonella*, identified by antennal transcriptome analysis. PloS one, 7(2).

Berg, B.G., Galizia, C. G., Brandt, R., Mustaparta, H., 2002. Digital Atlases of the Antennal Lobe in Two Species of Tobacco Budworm Moths, the Oriental *Helicoverpa assulta* (Male) and the American *Heliothis virescens* (Male and Female). The Journal Of Comparative Neurology 446:123–134.

Dong, J., Song, Y., Li, W., Shi, J., Wang, Z. 2016. Identification of putative chemosensory receptor genes from the *Athetis dissimilis* antennal transcriptome. PLoS One, 11(1).

Grosse-Wilde, E., Kuebler, L.S., Bucks, S., Vogel, H., Wicher, D., Hansson, B.S., 2011. Antennal transcriptome of *Manduca sexta*. Proceedings of the National Academy of Sciences, 108(18), 7449-7454.

Gu, S. H., Sun, L., Yang, R.N., Wu, K.M., Guo, Y.Y., Li, X.C., Zhou, J.J., Zhang, Y.J. 2014. Molecular characterization and differential expression of olfactory genes in the antennae of the black cutworm moth *Agrotis ipsilon*. PloS one, 9(8).

Greiner, B., Gadenne, C., Anton, S. 2004. Three‐dimensional antennal lobe atlas of the male moth, *Agrotis ipsilon*: A tool to study structure–function correlation. Journal of Comparative Neurology, 475(2), 202-210.

Jacquin-Joly, E., Legeai, F., Montagné, N., Monsempes, C., François, M. C., Poulain, J., Gavory, F., Walker Ⅲ, W.B., Hansson, B.S. Larsson, M. C. 2012. Candidate chemosensory genes in female antennae of the noctuid moth *Spodoptera littoralis*. International journal of biological sciences, 8(7), 1036.

Koenig, C., Hirsh, A., Bucks, S., Klinner, C., Vogel, H., Shukla, A., Mansfield, J.H., Morton, B., Hansson, B.S., Grosse-Wilde, E. 2015. A reference gene set for chemosensory receptor genes of *Manduca sexta*. Insect Biochemistry and Molecular Biology, 66, 51-63.

Liu, Y., Gu, S., Zhang, Y., Guo, Y., Wang, G. 2012. Candidate olfaction genes identified within the *Helicoverpa armigera* antennal transcriptome. PloS one, 7(10).

Liu, N. Y., Xu, W., Papanicolaou, A., Dong, S. L., Anderson, A. 2014. Identification and characterization of three chemosensory receptor families in the cotton bollworm *Helicoverpa armigera*. BMC Genomics, 15(1), 597.

Poivet, E., Gallot, A., Montagné, N., Glaser, N., Legeai, F., Jacquin-Joly, E. 2013. A comparison of the olfactory gene repertoires of adults and larvae in the noctuid moth *Spodoptera littoralis*. PloS one, 8(4).

Rospars, J.P., Hildebrand, J.G. 2000. Sexually dimorphic and isomorphic glomeruli in the antennal lobes of the sphinx moth *Manduca sexta*. Chemical senses, 25(2), 119-129.

Sadek, M.M., Hansson, B.S., Rospars, J.P., Anton, S., 2002. Glomerular representation of plant volatiles and sex pheromone components in the antennal lobe of the female *Spodoptera littoralis*. The Journal of Experimental Biology 205, 1363–1376.

Skiri, H.T. , Helge, R., Berg, B.G., Mustaparta, H., 2005. Consistent organization of glomeruli in the antennal lobes of related species of heliothine moths. Journal of Comparative Neurology, 491(4).

Trona, F., Anfora, G., Bengtsson, M., Witzgall, P., Ignell, R. 2010. Coding and interaction of sex pheromone and plant volatile signals in the antennal lobe of the codling moth *Cydia pomonella*. Journal of Experimental Biology, 213(24), 4291-4303.

Wan, X., Li, J., Lao, C., Du, Y.J. 2015. Antennal lobe neurons of *Spodoptera litura* (Lepidoptera: Noctuidae) and their responses to plant odors and sex pheromones. Acta Entomologica Sinica, 58(3), 223-236.

Xu, W., Papanicolaou, A., Liu, N.Y., Dong, S.L., Anderson, A. 2015. Chemosensory receptor genes in the Oriental tobacco budworm *Helicoverpa assulta*. Insect molecular biology, 24(2), 253-263.

Yang, S., Cao, D., Wang, G., Liu, Y. 2017. Identification of genes involved in chemoreception in *Plutella xyllostella* by antennal transcriptome analysis. Scientific reports, 7(1), 1-16.

Zhang, J., Wang, B., Dong, S., Cao, D., Dong, J., Walker, W. B., Liu, Y,. Wang, G. 2015. Antennal transcriptome analysis and comparison of chemosensory gene families in two closely related noctuidae moths, *Helicoverpa armigera* and *H. assulta*. PloS one, 10(2).

Zhao, X.C., Chen, Q.Y., Guo, P., Xie, G.Y., Tang, Q.B., Guo, X.R., Berg, B. G. 2016a. Glomerular identification in the antennal lobe of the male moth *Helicoverpa armigera*. Journal of Comparative Neurology, 524:2993–3013.

Zhao, X.C., Ma, B.W., Berg, B.G., Xie, G.Y., Tang, Q.B., Guo, X.R. 2016b. A global-wide search for sexual dimorphism of glomeruli in the antennal lobe of female and male *Helicoverpa armigera*. Scientific reports, 6(1), 1-9.
